# Attitudes, knowledge, and perceptions of dentists and dental students toward artificial intelligence: a systematic review

**DOI:** 10.1016/j.jtumed.2023.12.010

**Published:** 2024-01-12

**Authors:** Mahmood Dashti, Jimmy Londono, Shohreh Ghasemi, Zohaib Khurshid, Farshad Khosraviani, Negar Moghaddasi, Muhammad S. Zafar, Delband Hefzi

**Affiliations:** a Department of Dentistry, School of Dentistry, Shahid Beheshti University of Medical Sciences, Tehran, Iran; b Department of Oral and Maxillofacial Surgery, Board Certified Prosthodontist, FACP, Professor and Director of the Prosthodontics Residency Program and the Ronald Goldstein Center for Esthetics and Implant Dentistry, Dental College of Georgia at Augusta University, Augusta, GA, USA; c MSc of Trauma and Craniofacial Reconstruction, Queen Mary College, London, UK; d Department of Prosthodontics and Dental Implantology, College of Dentistry, King Faisal University, Al-Ahsa, Saudi Arabia; e Center of Excellence for Regenerative Dentistry, Department of Anatomy, Faculty of Dentistry, Chulalongkorn University, Bangkok, Thailand; f Department of Dentistry, UCLA School of Dentistry, Los Angeles, CA, USA; g Department of Dentistry, College of Dental Medicine, Western University of Health Sciences, CA, USA; h Department of Restorative, Dentistry, Taibah University, Almadinah Almunawwarah, KSA; i Department of Dental Materials, Islamic International Dental College, Riphah International University, Islamabad, Pakistan; j Department of Dentistry, School of Dentistry, Tehran University of Medical Science, Tehran, Iran

**Keywords:** الذكاء الاصطناعي, التعلم العميق, تعلم الآلة, طب الأسنان, طلاب طب الأسنان, ممارس طب الأسنان, Artificial intelligence, Deep learning, Dental practitioner, Dental students, Dentistry, Machine learning

## Abstract

**Objectives:**

This research was aimed at assessing comprehension, attitudes, and perspectives regarding artificial intelligence (AI) in dentistry. The null hypothesis was a lack of foundational understanding of AI in dentistry.

**Methods:**

This systematic review following Preferred Reporting Items for Systematic Reviews and Meta-Analyses (PRISMA) guidelines was conducted in May 2023. The eligibility criteria included cross-sectional studies published in English until July 2023, focusing solely on dentists or dental students. Data on AI knowledge, use, and perceptions were extracted and assessed for bias risk with the Joanna Briggs Institute checklist.

**Results:**

Of 408 publications, 22 relevant articles were identified, and 13 studies were included in the review. The average basic AI knowledge score was 58.62 % among dental students and 71.75 % among dentists. More dental students (72.01 %) than dentists (62.60 %) believed in AI's potential for advancing dentistry.

**Conclusions:**

Thorough AI instruction in dental schools and continuing education programs for practitioners are urgently needed to maximize AI's potential benefits in dentistry. An integrated PhD program could drive revolutionary discoveries and improve patient care globally. Embracing AI with informed understanding and training will position dental professionals at the forefront of technological advancements in the field.

## الملخص

**أهداف البحث:**

يهدف هذا البحث إلى تقييم فهم ومواقف ووجهات نظره أطباء وطلبة طب الأسنان تجاه الذكاء الاصطناعي في طب الأسنان، مما يتحدى الفرضية الصفرية المتمثلة في افتقارهم إلى الفهم الأساسي.

**طرق البحث:**

أجرت الدراسة مراجعة منهجية وفقا لإرشادات بريزما، اعتبارا من مايو 2023. تضمنت الدراسات المؤهلة مقالات مقطعية نشرت باللغة الإنجليزية حتى يوليو 2023 ، مع التركيز فقط على أطباء الأسنان أو طلاب طب الأسنان. تم استخراج البيانات المتعلقة بالمعرفة الذكاء الاصطناعي والاستخدام والتصورات وتقييمها لمخاطر التحيز باستخدام قائمة مراجعة معهد جوانا بريجز.

**النتائج:**

من بين 408 منشورات، تم تحديد 22 مقالة ذات صلة، وتم تضمين 13 دراسة في المراجعة. كان متوسط المعرفة الأساسية الذكاء الاصطناعي لطلاب طب الأسنان 58.62٪ ، بينما كانت المعرفة الأولية لأطباء الأسنان 71.75٪. أظهر طلاب طب الأسنان إيمانا أعلى (72.01٪) بإمكانات الذكاء الاصطناعي للتقدم في طب الأسنان مقارنة بأطباء الأسنان (62.60٪).

**الاستنتاجات:**

هناك حاجة ماسة إلى تعليم الذكاء الاصطناعي في كليات طب الأسنان، إلى جانب برامج التعليم المستمر للممارسين، لتعظيم الفوائد المحتملة للذكاء الاصطناعي في طب الأسنان. يمكن لبرامج الدكتوراه المتكاملة أن يقود الاكتشافات وتحسن رعاية المرضى على مستوى العالم. إن احتضان الذكاء الاصطناعي مع الفهم المستنير والتدريب سيضع المتخصصين في طب الأسنان في طليعة التطورات التكنولوجية في هذا المجال.

## Introduction

Although the term “artificial intelligence” (AI) emerged in the 1950s, AI has recently become a valuable tool.^1^^,^^2^ AI involves using machines and algorithms to replicate the human brain and perform tasks typically requiring human effort.^1^^,^^3^ Over time, three major AI subcategories—machine learning, deep learning, and conventional neural networks—evolved and have been applied to the analysis of complex data from various sources.^3^^,^^4^

A surge in AI implementation has been observed across multiple industries, such as manufacturing, engineering, the stock market, information communications, medicine, and dentistry.^4^^,^^5^ In dentistry, AI applications have found success in a wide range of areas, including facial growth analysis, cephalometric landmark tracking, implant placement, detection of caries, bone loss, periapical disease, oral cancer, identification of radiolucent and cystic lesions, tooth color selection, and tooth-root morphology.^3^^,^^4^^,^^6^ In addition, AI has been found to outperform human experts in diagnosing pigmented skin lesions.^4^^,^^7^

However, dental AI applications remain in early stages and are not universally used.^7^ Several studies have indicated that dentists and dental students have not fully grasped the potential of AI.^1^, ^2^, ^3^, ^4^ Diverse perspectives exist regarding the effects of AI on daily life, ranging from optimistic to pessimistic.^8^ Nevertheless, the promising future of AI in dentistry^1^ is evident, given the substantial progress in AI applications within industry. Perception—referring to the ability to understand—involves the mental process of acquiring knowledge and understanding the meaning of information, ideas, or concepts. Attitudes refer to individuals’ evaluative judgments or feelings toward people, objects, ideas, or situations; these complex mental states involve a combination of beliefs, emotions, and behavioral tendencies.

Previous systematic reviews evaluating dentists' and dental students’ knowledge regarding AI and the importance of using AI in dentistry are lacking. Therefore, this research was aimed at assessing the comprehension, attitudes, and perspectives of dentists and dental students regarding AI and its use in dentistry. The null hypothesis was that dentists and dental students lack a foundational understanding and perspective of AI and its applications in dentistry.

## Materials and Methods

**Figure 1:**
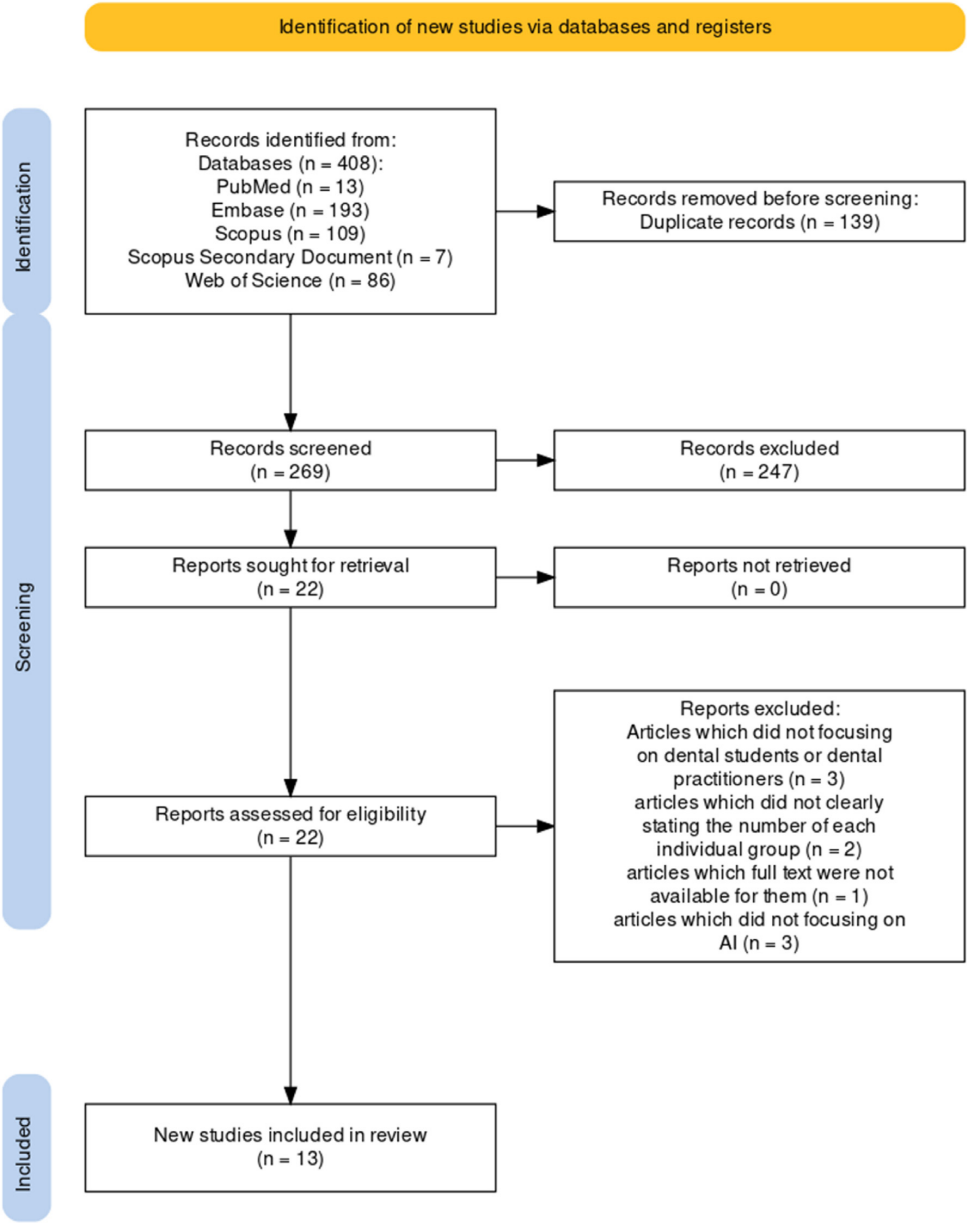
PRISMA flowchart for article selection.

This systematic review followed the Preferred Reporting Items for Systematic Reviews and Meta-Analyses (PRISMA) guidelines^9^ for extracting, selecting, and screening research articles (Figure 1). The population, intervention, control, and outcomes (PICO) question was as follows:

Population: The population consisted of dentists and dental students.
Intervention: The intervention involved assessing their comprehension, attitudes, and perspectives toward AI in dentistry.
Control: The control group consisted of dentists and dental students who had not been introduced to AI.
Outcomes: The outcomes included the attitudes, knowledge, and perceptions of dentists and dental students toward AI and its use in dentistry.

The primary research question was: what are the attitudes, knowledge, and perceptions of dentists and dental students toward the AI and its use in dentistry?

### Eligibility criteria

The following criteria were used to determine which studies were included in this systematic review: (1) cross-sectional studies; (2) articles focusing solely on dentists or dental students; (3) articles published until July 2023; and (4) articles published only in English. We considered only research meeting these criteria.

Studies were excluded if they: (1) had already undergone a systematic review, meta-analysis, or scoping review; (2) had been published in a language other than English; (3) did not focus solely on dentists or dental students; or (4) lacked accurate survey results from dentists or dental students.

### Research strategy and screening

In May 2023, five databases—PubMed, Web of Science, Embase, Scopus, and Scopus Secondary—were used to systematically search and screen research articles for studies published until July 2023. For screening and selection of articles for inclusion, we used the PRISMA systematic review and meta-analysis standards. The keywords used for each specific database are listed in Table 1; the total search was intended to assess articles from various fields.

**Table 1:** Keywords used to search each database.

Database	Keyword	Result
PubMed	((“knowledge” OR “belief” OR “beliefs” OR “believe” OR “perspective” OR “opinion” OR “Attitude” OR “perception”) AND (“artificial intelligence” OR “Machine Learning” [Mesh] OR “Neural Networks, Computer” [Mesh] OR “Supervised Machine Learning” [Mesh] OR “Deep Learning” [Mesh] OR “Unsupervised Machine Learning” [Mesh])) AND (“Dentists” [Mesh] OR “Dental Staff” [Mesh] OR “Faculty, Dental” [Mesh] OR “Students, Dental” [Mesh])	13
Embase	(“supervised machine learning” OR “machine learning”/exp OR “machine learning” OR “supervised machine learning”/exp OR “unsupervised machine learning”/exp OR “deep learning”/exp OR “deep learning” OR “unsupervised machine learning” OR “artificial neural network”/exp OR “artificial neural network” OR “artificial intelligence”) AND (“knowledge”/exp OR “knowledge” OR “attitude”/exp OR “attitude” OR “health personnel attitude”/exp OR “health personnel attitude” OR “belief”/exp OR “belief” OR “beliefs about medicines questionnaire”/exp OR “beliefs about medicines questionnaire” OR “beliefs”/exp OR “beliefs” OR “believe” OR “perspective”/exp OR “perspective” OR “opinion” OR “perception”/exp OR “perception”) AND (“dentist”/exp OR “dentist” OR “dentists”/exp OR “dentists” OR “dental staff”/exp OR “dental staff” OR “dental education”/exp OR “dental education” OR “dental faculty”/exp OR “dental faculty” OR “dental student”/exp OR “dental student” OR “dental students”/exp OR “dental students” OR “dentistry”/exp)	193
Scopus	(TITLE-ABS-KEY (“Machine Learning”) OR TITLE-ABS-KEY (“Deep Learning”) OR TITLE-ABS-KEY (“Supervised Machine Learning”) OR TITLE-ABS-KEY (“Unsupervised Machine Learning”) OR TITLE-ABS-KEY (“Neural Network”) OR TITLE-ABS-KEY (“artificial intelligence”)) AND (TITLE-ABS-KEY (“knowledge”) OR TITLE-ABS-KEY (“belief”) OR TITLE-ABS-KEY (“beliefs”) OR TITLE-ABS-KEY (“believe”) OR TITLE-ABS-KEY (“perspective”) OR TITLE-ABS-KEY (“opinion”) OR TITLE-ABS-KEY (“Attitude”) OR TITLE-ABS-KEY (“perception”)) AND (TITLE-ABS-KEY (“Dentist”) OR TITLE-ABS-KEY (“Dentists”) OR TITLE-ABS-KEY (“Dental Staff”) OR TITLE-ABS-KEY (“Dental faculty”) OR TITLE-ABS-KEY (“Dental student”) OR TITLE-ABS-KEY (“Dental students”))	109
Scopus Secondary	(TITLE-ABS-KEY (“Machine Learning”) OR TITLE-ABS-KEY (“Deep Learning”) OR TITLE-ABS-KEY (“Supervised Machine Learning”) OR TITLE-ABS-KEY (“Unsupervised Machine Learning”) OR TITLE-ABS-KEY (“Neural Network”) OR TITLE-ABS-KEY (“artificial intelligence”)) AND (TITLE-ABS-KEY (“knowledge”) OR TITLE-ABS-KEY (“belief”) OR TITLE-ABS-KEY (“beliefs”) OR TITLE-ABS-KEY (“believe”) OR TITLE-ABS-KEY (“perspective”) OR TITLE-ABS-KEY (“opinion”) OR TITLE-ABS-KEY (“Attitude”) OR TITLE-ABS-KEY (“perception”)) AND (TITLE-ABS-KEY (“Dentist”) OR TITLE-ABS-KEY (“Dentists”) OR TITLE-ABS-KEY (“Dental Staff”) OR TITLE-ABS-KEY (“Dental faculty”) OR TITLE-ABS-KEY (“Dental student”) OR TITLE-ABS-KEY (“Dental students”))	7
WOS	((TS = (“Machine Learning” OR “Deep Learning” OR “Neural Networks” OR “Supervised Machine Learning” OR “artificial intelligence” OR “Unsupervised Machine Learning”)) AND TS = (“knowledge” OR “belief” OR “beliefs” OR “believe” OR “perspective” OR “opinion” OR “Attitude” OR “perception”)) AND TS = (“Dentist” OR “Dentists” OR “Dental Staff” OR “Dental faculty” OR “Dental student” OR “Dental students”)	86

Two reviewers (M.D. and D.H.) separately evaluated the titles and abstracts, and a third reviewer (J.L.) settled disagreements. All included studies were required to fully satisfy the inclusion criteria and to have full text available.

### Data collection and synthesis

Table 2 presents the information obtained from the published studies. The extracted information was based on study characteristics and included the authors, publication year, country of study, number of dental students or dental practitioners, gender, basic knowledge of AI and the use of AI in dentistry, belief that AI will result in significant advancements in dentistry, and belief that AI will eventually replace dentists. The above characteristics were used to demonstrate and evaluate perceptions and knowledge among dentists and dental students (see Table 2).

**Table 2:** Data extraction table.

Author/year	Country	DS/DP	Gender	Basic knowledge regarding AI	AI use in dentistry	AI will lead to major advances in dentistry	AI will replace dentists in the future
Aboalshamat et al., 2022^6^	KSA	DS = 230 DP = 159	M = 111/F = 278	Yes = 44.5 %	Yes = 42.2 %	Yes = 75 %	Yes = 49.1 %
AlAhmari, 2022^10^	KSA	DS = 218	M = 116/F = 102	Yes = 22 %	Yes = 37 %	Yes = 74 %	Yes = 36 %
Asmatahsain et al., 2021^1^	India	DS = 270	M = 49/F = 221	Yes = 89.63 %	Yes = 43.7 %	Yes = 77.04 %	Yes = 20.74 %
Jethlia et al., 2022^2^	KSA	DS = 102 DP = 98	M = 100/F = 100	DS Yes = 72.5 % DP Yes = 75.5 %	DS Yes = 62.7 % DP Yes = 66.3 %	DS Yes = 64.7 % DP Yes = 62.2 %	DS Yes = 17.6 % DP Yes = 12.2 %
Keser et al., 2021^3^	Turkey	DS = 140	M = 55/F = 85	Yes = 60 %	Yes = 39.3 %	Yes = 37.9 %	
Khanagar et al., 2021^11^	KSA	DS = 423	M = 215/F = 208	Yes = 49.9 %	Yes = 44.2 %	Yes = 86.5 %	
Seram et al., 2021^7^	India	DS = 279	M = 101/F = 178	Yes = 59.85 %	Yes = 63 %	Yes = 61.6 %	
Sur et al., 2020^4^	India	DP = 250		Yes = 68 %	Yes = 42 %	Yes = 63 %	
Thulasi et al., 2022^12^	India	DS = 140 DP = 60	M = 99/F = 111		Yes = 34.5 %	Yes = 40 %	Yes = 29.5 %
Vamshi Ram et al., 2022^13^	India	DS = 100		Yes = 67 %	Yes = 84 %		
Yuzbasioglu, 2021^8^	Turkey	DS = 1103	M = 453/F = 650	Yes = 48.4 %	Yes = 78.8 %	Yes = 85.7 %	Yes = 28.6 %
Akhtar et al., 2022^14^	Pakistan	DS = 355	M = 95/F = 260	Yes = 58.3 %	Yes = 41.4 %	Yes = 74.7 %	Yes = 33.8 %
Karan-Romero et al., 2023^15^	Peru	DS = 200	M = 102/F = 98			Yes = 86 %	Yes = 34 %

DS: Dental student; DP: dental practitioner; M: male; F: female.

### Quality assessment

We used the Joanna Briggs Institute checklist^16^ for evaluating the risk of bias regarding technique and reporting findings. The checklist consists of nine elements: inclusion criteria, description of the study participants and context, exposure measurement, objectivity, use of standard criteria, confounding variables, handling of confounding variables, outcomes measured, and use of proper statistical analysis. In the event of disagreement between the first two authors, a third author (S.G.) rated the risk of bias as “yes,” “no,” or “not clear” (Table 3).

**Table 3:** Joanna Briggs Institute Quality Assessment.

	1. Was the sample representative of the target population?	2. Were study participants recruited in an appropriate way?	3. Was the sample size adequate?	4. Were the study participants and setting described in detail?	5. Was the data analysis conducted with sufficient coverage of the identified sample?	6. Were objective, standard criteria used for the measurement of the condition?	7. Was the condition measured reliably?	8. Was appropriate statistical analysis performed?	9. Are all important confounding factors and subgroup differences identified and accounted for?	10. Were subpopulations identified with objective criteria?	Overall appraisal
Khalid T. Aboalshamat et al., 2022	Yes	Yes	Yes	Yes	Yes	No	Yes	Yes	No	Yes	Include
Fatemah AlAhmari et al., 2022	Yes	Yes	Yes	Yes	NA	Yes	NA	Yes	No	Yes	Include
Mohammed Asmatahasin et al., 2021	Yes	Yes	No	Yes	No	No	NA	yes	No	Yes	Include
Ankur Jethlia et al., 2022	Yes	Yes	Yes	Yes	NA	Yes	NA	Yes	No	Yes	Include
Filiz Namdar Pekiner et al., 2021	Yes	No	Yes	Yes	NA	No	NA	Yes	No	Yes	Include
Sanjeev Khanagar et al., 2021	Yes	Yes	Yes	Yes	Yes	No	Yes	Yes	No	Yes	Include
Tampha Seram et al., 2021	Yes	Yes	No	Yes	Yes	Yes	NA	Yes	No	Yes	Include
Jaideep Sur et al., 2020	Yes	Yes	Yes	Yes	Yes	No	NA	Yes	No	Yes	Include
M. Shiva Thulasi et al., 2022	Yes	Yes	Yes	Yes	Yes	No	Yes	Yes	No	Yes	Include
VAMSHI RAM V et al., 2022	No	No	Yes	Yes	No	Yes	Yes	Yes	No	Yes	Include
Emir Yüzbaşıoğlu et al., 2021	Yes	Yes	Yes	Yes	Yes	No	Yes	Yes	No	Yes	Include
Milan Karan-Romero et al., 2023	Yes	Yes	Yes	Yes	Yes	No	Yes	Yes	No	Yes	Include
Hira Akhtar et al.	No	Yes	Yes	Yes	NA	Yes	NA	Yes	No	Yes	Include

## Results

### Identified studies

From the aforementioned databases, the search yielded produced 408 research articles on the attitudes, knowledge, and perceptions of dental practitioners and students regarding AI and its applications in dentistry. A total of 22 articles were found to be relevant, reliable, and consistent with the goals of the study, on the basis of the inclusion criteria. On the basis of the exclusion criteria, 9 of the 22 articles were excluded, and 13 of the remaining articles met the standards and were added to the systematic review, thus resulting in the inclusion of 10 studies in the analysis (Figure 1).

The nine excluded articles comprised three articles not focusing on dental students or dental practitioners, two articles not clearly stating the number of each individual group, one article for which full text was unavailable, and three articles not focusing on AI.

### Summary of identified studies

Among the 408 studies selected, 13 articles were included in the data extraction.^1^, ^2^, ^3^, ^4^^,^^6^, ^7^, ^8^^,^^10^, ^11^, ^12^, ^13^, ^14^, ^15^ These studies were cross-sectional surveys conducted in KSA, India, Turkey, Pakistan, and Peru, thus indicating that primarily Asian countries have been focusing on this subject. Furthermore, the studies included those examining attitudes, knowledge, and perceptions among dental students and dental practitioner regarding AI; the outcomes were reported as percentages of answers regarding basic knowledge of AI, AI use in dentistry, a belief that AI will lead to major advances in dentistry, and a belief that AI will replace dentists in the future (Table 2).

### Risk of bias

Most studies clearly described the study group and setting in detail (n = 13). However, none of the studies examined confounding factors. Only six studies reported the validity and reliability of the questionnaire without any bias, whereas the validity for the other studies was unclear. Most studies clearly described the study participants and settings (n = 10); because the studies were observational, use of only descriptive statistics was appropriate and was applied in all studies.

Ten studies were included in the analysis.^1^, ^2^, ^3^^,^^7^^,^^8^^,^^10^^,^^11^^,^^13^, ^14^, ^15^ Only studies focusing on the level of knowledge, attitudes, and perceptions of dental students were included. High inter-examiner agreement (k = 0.92) was subsequently reached between the researchers.

### Knowledge regarding AI

**Figure 2:**
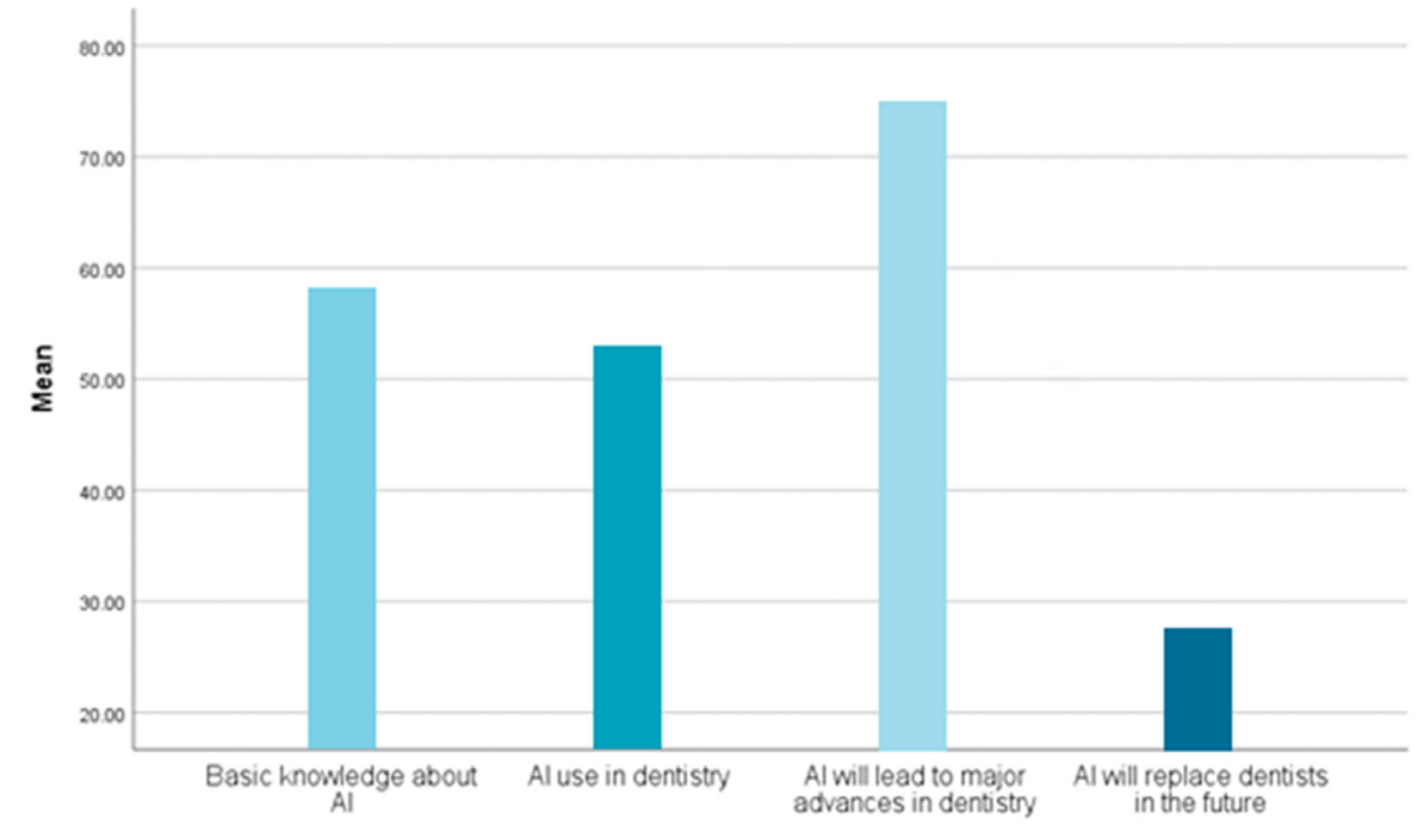
Comparison of the means of the reported values for dental students in the included studies.

The average basic knowledge score of dental students regarding AI was 58.62 ± 18.53 %, and the average use of AI in dentistry by dental students was 54.90± 17.78 %. On average, 72.01± 15.63 % of dental students believed that AI will lead to significant advancements in dentistry, whereas 28.45± 7.66 % believed that AI will replace dentists in the future (Figure 2).

**Figure 3:**
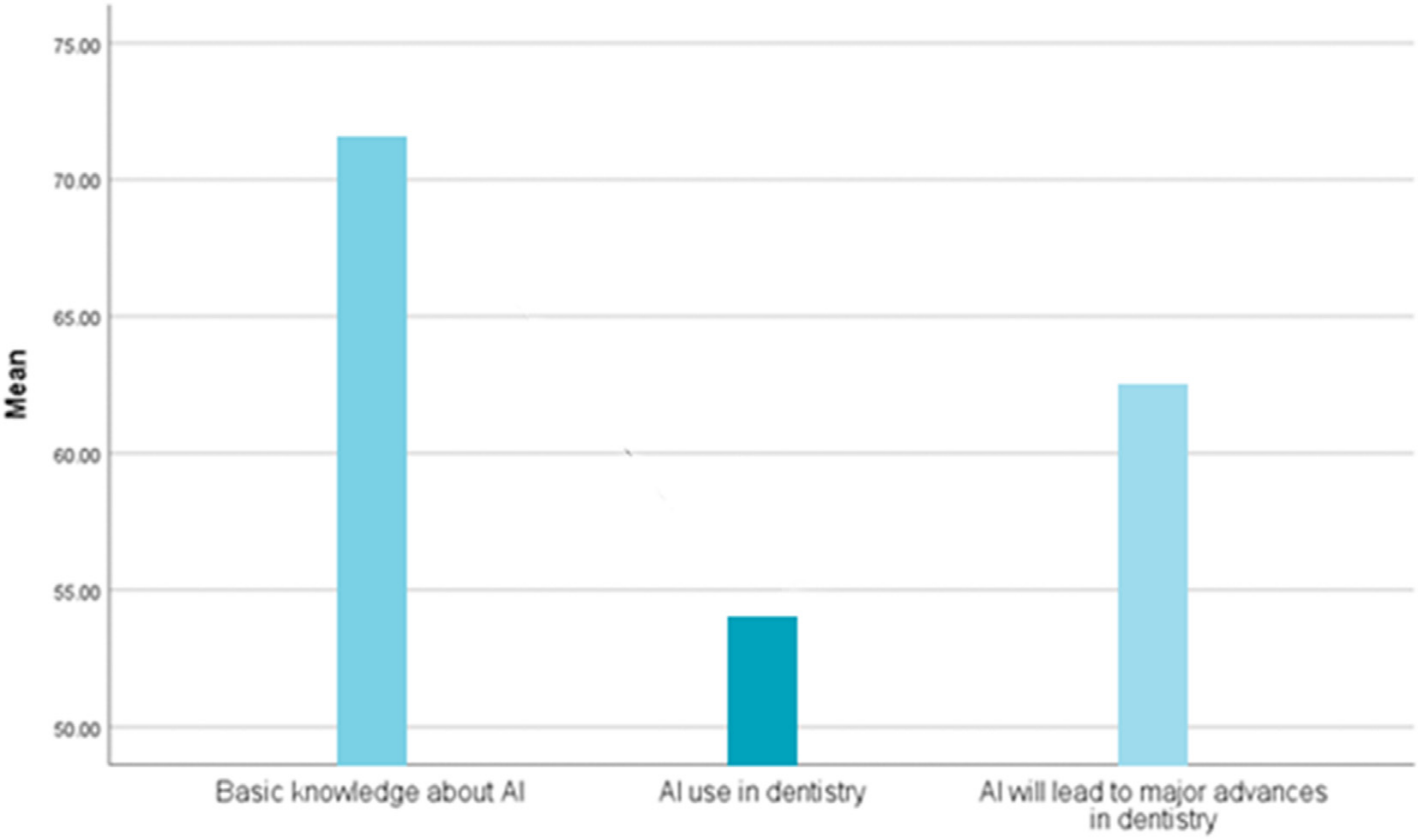
Comparison of the means of the reported values for dental practitioners in the included studies.

The average initial knowledge score of dentists regarding AI was 71.75 ± 5.30 %, and the average use of AI in dentistry by dentists was 54.15± 17.18 %. On average, 62.60 ± 0.56 % of dentists believed that AI will lead to substantial advancements in dentistry (Figure. 3).

**Figure 4:**
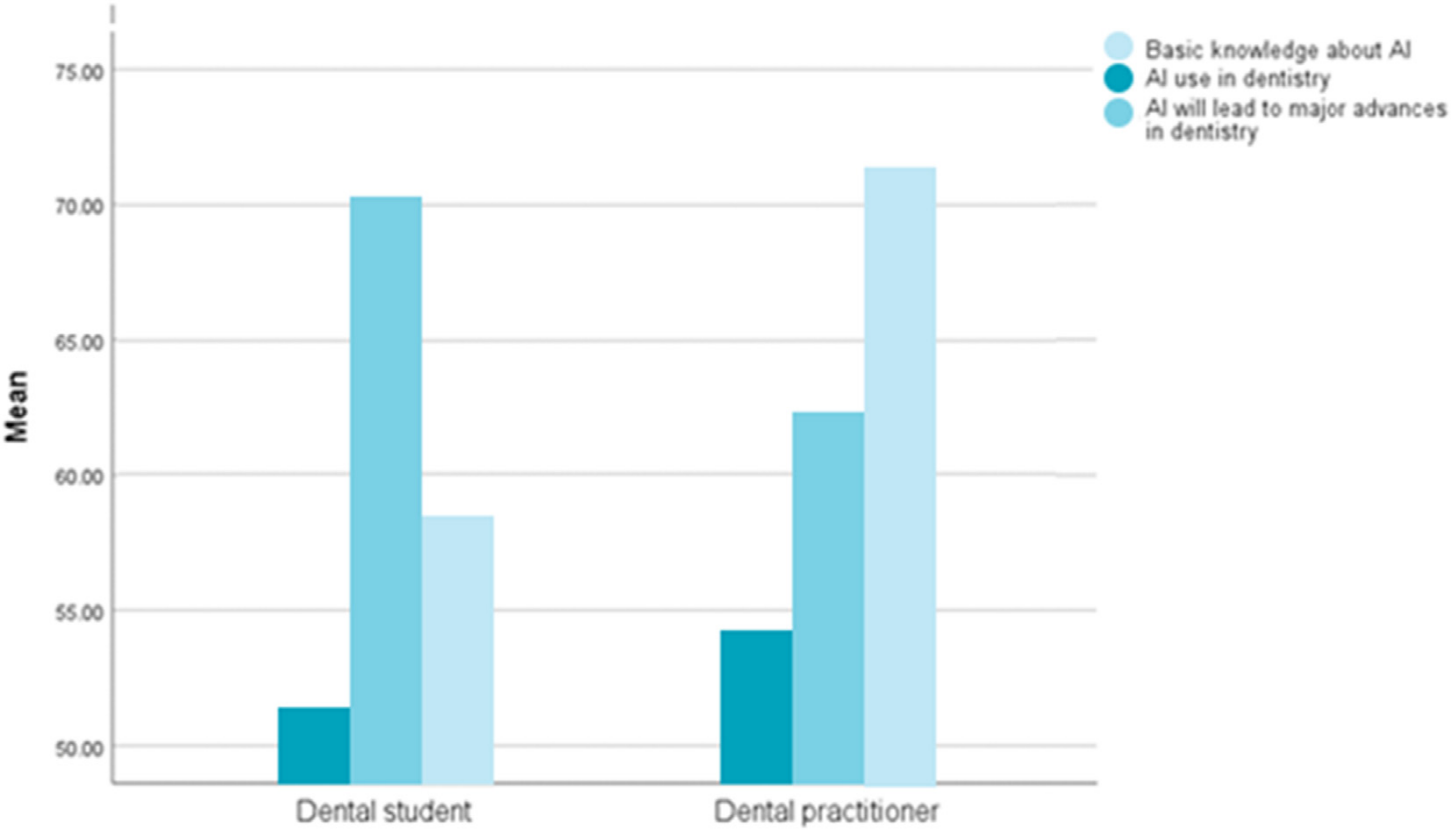
Comparison of the means of the reported values between dental students and dental practitioners in the included studies.

As shown in Figure 4, dental students had less initial knowledge of AI than dentists. Although dentists used AI in dentistry more than dental students, more dental students believed that AI is poised to enable substantial progress in dentistry.

## Discussion

Our study showed that dental students have an acceptable understanding of what AI is but less understanding of AI's applications in dentistry. Thus, dental schools must implement courses for dental students, dental practitioners, and even PhD students. However, a study by Roganvic et al. has shown a lack of knowledge regarding AI among dental undergraduate students.^17^

### The need for thorough AI instruction at dental schools

Dental schools must incorporate thorough AI instruction into their courses to bridge the knowledge gaps between dentists and dental students.^18^ AI has a broad range of applications in education and healthcare, and it may enhance and supplement human labor. Adoption of AI might facilitate existing operations and future innovation, while improving educational opportunities and healthcare services.^19^ Future dental professionals will be more prepared to embrace the possibilities of AI-driven advancements if they receive a sound foundation in AI knowledge early in their training, thus raising the standards for dental practice.

### Curriculum improvement

Dental schools should add courses on AI that address foundational ideas, AI algorithms, and dental applications. Students' practical abilities in using AI technologies for patient diagnosis and treatment planning could also be improved through hands-on workshops and simulation-based training. A core curriculum on oral and dental AI may help increase oral and dental healthcare providers’ AI literacy, thus allowing them to critically appraise AI and be aware of current advancements in the field.^20^

### Collaboration with AI professionals

To create cutting-edge AI applications well suited to dentistry, dental institutions can work with academics and AI professionals. This multidisciplinary approach helps dentistry students investigate AI's various capabilities within the dental industry and spurs innovation. AI has the potential to revolutionize the field of dentistry by serving as a supplemental tool to improve the precision of diagnosis, treatment planning, and prediction of treatment results.^21^

### Ethics and privacy considerations

To enable ethical and responsible AI use in dentistry offices, AI training should also cover ethical issues, patient privacy, and data security. Female dentists, compared with male dentists, have been reported to have greater awareness of ethical concerns associated with the integration of AI in dental practices.^17^

### Continuing education among dental practitioners

Continued learning is necessary to keep dental professionals abreast of the most recent developments and best practices, given AI's rapid advancement. Dental professionals must continually refresh their knowledge and abilities to remain at the cutting edge of dental breakthroughs.^19^ Dentistry practitioners may become familiar with the most recent AI technology and best practices through continuing education (CE) courses designed for AI applications. These courses will make practitioners better equipped to integrate AI into their everyday practices, thus ensuring the best possible patient results and improving dental care.^20^ Using CE programs that emphasize AI applications in dentistry would provide the following advantages:

### Fostering a culture of continuous professional development

CE courses enable dental professionals to remain interested and engaged, thus promoting a culture of lifelong learning.

### Adoption of advanced techniques

AI-driven tools and technologies can increase diagnostic accuracy, expedite operations, and improve treatment results.^21^ Dental professionals may gain practical experience in cutting-edge treatments through CE courses.

### Improving patient care

Modern AI expertise enables dental professionals to provide state-of-the-art procedures, individualized care regimens, and enhanced patient experiences. AI can also help expedite responses to interventions; streamline workflows; allow employees to spend less time on lengthy processes and manual tasks, and ultimately dedicate more hours to patient care and efficient hospital administration; and decrease stress among physicians and medical staff.^22^

### Addressing challenges and concerns

To promote education and awareness of AI's role in dentistry practice, CE courses should address the worries and misunderstandings surrounding AI. These courses can provide dentists and dental professionals with the necessary knowledge to understand how AI is being integrated into dentistry, its potential benefits, and the limitations and ethical considerations associated with its use. By addressing these concerns and misconceptions, CE courses can help build trust in AI technologies and encourage their responsible and effective implementation in dental practice, thus ultimately improving patient care and outcomes.^23^

### Establishing integrated PhD programs

Integrated PhD programs should be established with an emphasis on AI applications in dentistry to enhance AI-driven dental research and innovation. With the help of such initiatives, dental professionals and AI experts may work more closely across disciplines in advancing ground-breaking research and creating advanced AI tools suited for dental practice.

### Collaborative research

Research initiatives involving dental practitioners and AI specialists have the potential to yield ground-breaking findings and advancements. Collaborative research and educational programs can bridge the gap between the dental and technological domains, thereby fostering a synergistic approach that maximizes AI's potential to assist both dental practitioners and patients.^24^

### Advanced specialization

PhD programs can help dentists focus on certain AI applications in dentistry, such as oral pathology, treatment planning, or prediction of patient outcomes.

### Changing dentistry practice

PhD graduates may help improve patient care by creating inventive AI-driven solutions that will reshape the dentistry industry.

Several limitations of this study should be acknowledged. First, the regional concentration of some studies, particularly in KSA and India, might restrict the generalizability of findings to a more global context. Additionally, the exclusion of studies in languages other than English might potentially have introduced a language bias.

## Conclusion

The research results discussed herein highlight the urgent need for thorough AI instruction in dental schools to enable aspiring dentists to fully realize the benefits of AI in dentistry. Additionally, to help practitioners keep abreast of the rapid breakthroughs in AI, CE courses for dental professionals must be established. Moreover, integrated PhD programs emphasizing dental AI applications may lead to revolutionary discoveries and usher the dental industry into a new age of cutting-edge patient care. Dental professionals may continue to thrive at the forefront of technologic advancement and provide better outcomes for patients worldwide by using AI, building on a foundation of education, understanding, and solid training.

## Source of funding

This research did not receive any specific grant from funding agencies in the public, commercial, or not-for-profit sectors.

## Conflict of interest

We have no conflicts of interest to declare.

## Ethical approval

There are no ethical issues to declare.

## Authors contributions

M.D.: Conception and design of study, Acquisition of data, Drafting of article and/or critical revision, Final approval of manuscript. J.L.: Conception and design of study, Final approval of manuscript, Analysis of data. S.G.: Acquisition of data, Analysis of data, Drafting of article and/or critical revision, Final approval of manuscript. Z.K.: Conception and design of study, Analysis of data, Drafting of article and/or critical revision, Final approval of manuscript. F.K.: Conception and design of study, Acquisition of data, Drafting of article and/or critical revision, Final approval of manuscript. N.M.: Analysis of data, Drafting of article and/or critical revision, Final approval of manuscript. M.S.Z.: Conception and design of study, Drafting of article and/or critical revision, Final approval of manuscript. D.H.: Final approval of manuscript, Analysis of data, Drafting of article and/or critical revision. All authors have critically reviewed and approved the final draft and are responsible for the content and similarity index of the manuscript.
